# The Cytosolic Domain of Fis1 Binds and Reversibly Clusters Lipid Vesicles

**DOI:** 10.1371/journal.pone.0021384

**Published:** 2011-06-28

**Authors:** Robert C. Wells, R. Blake Hill

**Affiliations:** 1 Department of Biology, Johns Hopkins University, Baltimore, Maryland, United States of America; 2 Department of Chemistry, Johns Hopkins University, Baltimore, Maryland, United States of America; University of Oulu, Germany

## Abstract

Every lipid membrane fission event involves the association of two apposing bilayers, mediated by proteins that can promote membrane curvature, fusion and fission. We tested the hypothesis that Fis1, a tail-anchored protein involved in mitochondrial and peroxisomal fission, promotes changes in membrane structure. We found that the cytosolic domain of Fis1 alone binds lipid vesicles, which is enhanced upon protonation and increasing concentrations of anionic phospholipids. Fluorescence and circular dichroism data indicate that the cytosolic domain undergoes a membrane-induced conformational change that buries two tryptophan side chains upon membrane binding. Light scattering and electron microscopy data show that membrane binding promotes lipid vesicle clustering. Remarkably, this vesicle clustering is reversible and vesicles largely retain their original shape and size. This raises the possibility that the Fis1 cytosolic domain might act in membrane fission by promoting a reversible membrane association, a necessary step in membrane fission.

## Introduction

Peroxisomal and mitochondrial fission involve the cooperation of integral, peripheral, and soluble proteins to control organelle size, shape, and distribution [1]. The fission of these organelles appears to be executed by the same core machinery: a tail-anchored integral membrane protein, Fis1 [2]–[6], and a cytosolic dynamin-like mechanoenzyme, Dnm1 (yeast) or Drp1/Dlp1 (mammals) [7]–[9]. Other cytosolic proteins, such as Mdv1 and Caf4 in budding yeast, are thought to provide further control by modulating mechanoenzyme activity [10]. However, the exact mechanism by which these proteins regulate peroxisomal and mitochondrial fission is unknown, despite their importance in organelle homeostasis and human health [11]–[14].

The morphology of both peroxisomes and mitochondria in mammals is correlated with expression levels of Fis1, not the dynamin-like mechanoenzyme, suggesting an important role for Fis1 in fission regulation [6], [15]. Fis1 was initially discovered in yeast to be essential for the maintenance of mitochondrial homeostasis and is conserved in eukaryotes [2]–[5], [16]. This small, 17 kDa protein is uniformly localized on the cytosolic side of the mitochondrial outer membrane by a single-pass, C-terminal transmembrane domain, with a similar orientation on the peroxisomal surface [2], [6]. The Fis1 cytosolic domain is known to recruit other cytosolic factors that regulate or effect the fission process including Mdv1/Caf4 [9], [14], [17]–[24] and Dnm1 [25]. However, Fis1 also binds lipid vesicles altering membrane integrity enough to cause release of small, but not large, molecules contained within the vesicles [26]. This release of entrapped molecules occurs independently of other protein factors and may reflect an innate ability of Fis1 to alter membrane structure in a manner important for fission. Notably, release of vesicle contents did not require the transmembrane domain and can be induced by the cytosolic domain of Fis1 alone, which raises the possibility that the cytosolic domain itself plays a role in altering membrane structure, independently of its accepted role in protein recruitment.

Many soluble proteins regulate their activity through reversible interactions with membranes [27]–[29]. During membrane fusion, soluble domain interactions with membranes are essential for some SNARE-mediated fusion events, especially involving the SNARE Vam7p [30], [31] and synaptotagmin [32]–[35]. Whether these proteins directly deform membranes to mediate fusion is unclear, but it is likely that both membrane fusion and fission require proteins to stabilize high-energy membrane intermediates. Therefore, we would expect that the soluble domain may bind to synthetic membranes and alter their structures in a reversible manner. Here, we ask whether the soluble, cytosolic domain of Fis1 shares these properties. We find this domain alone is able to bind reversibly to lipid vesicles and determine what promotes this interaction. We identify that Fis1 undergoes a membrane-induced conformational change that clusters lipid vesicles. This clustering appears completely reversible, raising the possibility that Fis1 might help drive the association of two apposing bilayers that is required for membrane fission.

## Materials and Methods

### Protein Purification

DNA encoding the *S. cerevisiae* Fis1 gene lacking the C-terminal 27 residues (Fis1ΔTM) was subcloned into pET29b (EMD Biosciences) including a Tobacco etch virus (TEV) protease site and a C-terminal 6xHis tag. Plasmids were transformed into chemically competent *Escherichia coli* Rosetta cells (Novagen) and grown at 37°C in Luria broth with kanamycin (30 µg/mL) and chloramphenicol (34 µg/mL) to A_600_ of 0.7. Protein expression was induced by addition of 0.25 mM isopropyl 1-thio-β-d-galactopyranoside at 18°C and harvested 15–18 h later by centrifugation. The resulting cell pellets were resuspended in column buffer (20 mM Tris HCl, 250 mM NaCl, 1 mM dithiothreitol, 20 mM imidazole pH 7.4) containing protease inhibitors (Roche Applied Science). Cells were lysed with 4 passes through an Emulsiflex C3 (Avestin), DNase was added to 1 µg/mL and lysates were clarified by centrifugation. Protein was isolated from the resulting supernatant by affinity chromatography using Ni-Sepharose 6 Fast Flow beads (GE Healthcare), and eluted with a 100 mL linear gradient of column buffer with 500 mM imidazole. TEV protease was added at 1/100 molar ratio, put immediately into dialysis into column buffer at 4°C for 18 hours or until the protease reaction reached completion, determined by SDS-PAGE. Fis1ΔTM was separated from TEV protease and further purified on a Superdex-75 16/60 prep column (Amersham-Pharmacia). Samples were pooled and concentrated to ∼500 µM and stored at 4°C. Sample purity was checked by coomassie-stained SDS-PAGE and was typically greater than 95%.

### Vesicle Extrusion

All synthetic lipids were obtained from Avanti Polar Lipids (Alabaster, AL). The correct ratio of lipids were measured from chloroform stocks, mixed, and dried in a thin film under a stream of nitrogen. Dried films were lyophilized for at least 2 hours to remove excess chloroform and were resuspended in the required amount of deionized water to make a 12.6 mM solution of lipids. Lipid solutions were freeze-thawed in a dry ice/ethanol bath and 37°C water bath 11 times. Freeze-thawed solutions were extruded 11 times through a 100 nm nucleopore track etch membrane (Whatman), using an Avanti syringe extruder apparatus (Avanti Polar Lipids). Vesicle size, homogeneity and reproducibility were determined using dynamic light scattering and electron microscopy.

For spectroscopic characterizations, DOPC∶DOPG vesicles were made with 60% dioleoylphosphotidylcholine (DOPC) and 40% dioleoylphophotidylglycerol (DOPG), or different ratios as noted. Vesicles used for sedimentation included 1,2-dibromostearoyl-*sn*-glycero-3-phosphocholine (Br_4_DSPC) in place of DOPC, to allow for sedimentation at low centrifugal velocities and had 0.25% 1,2-dioleoyl-*sn*-glycero-3-phosphoethanolamine-N-(lissamine rhodamine B sulfonyl) (Rh-DOPE) doped for easy visualization of lipid pellets. Vesicles with mitochondrial membrane-like mixtures (Mitomix) were 48% tetrabrominated distearoylphosphatidylcholine (Br_4_DSPC), 28% phosphatidylethanolamine 16∶0–18∶1 (POPE), 10% phosphatidylinositol (PI), 10% dioleoylphosphatidylserine (DOPS), and 4% tetraoleoyl cardiolipin [36], [37].

### Vesicle Sedimentation

Vesicle sedimentation assays were performed with 5 µM protein in a 100 µL volume buffered with 20 mM potassium acetate and 20 mM potassium phosphate. Reactions were incubated with nutation for six hours at 25°C, and then subjected to centrifugation at increasing speeds (1,500, 6,000 and 18,000 rcf) for 30 minutes each [38], [39]. The top 80 µL was drawn off and diluted into 4× SDS loading buffer; the remaining 20 µL, which contained a mixture of soluble protein and trace lipids, was discarded. The lipid pellet was resuspended in 100 µL of buffer, and then diluted into 4× SDS loading buffer. 15 µL of each fraction was analyzed by SDS-PAGE and stained with coomassie G-250 Blue Silver stain for 18 hours and destained until background was negligible [40]. For samples measuring reversibility, the lipids were pelleted and resuspended by pipetting up and down with a 200 µL pipette, incubated for 2 hours, spun at three speeds and then prepared for SDS-PAGE as previously noted. Gels were scanned on a Canoscan flatbed scanner (Canon USA), band volume was calculated using Image Quant TL (Amersham), fraction bound was calculated using the equation: (V_pellet_)/(V_pellet_+V_supernatant_), where V_pellet_ and V_supernatant_ are the band volumes of the pellet and supernatant respectively. A Fis1ΔTM standard curve was made to assure the quantitation did not suffer from non-linear artifacts in the range of concentrations used, from the densitometry and was found to contain at most a 5% deviation from linearity. We corrected this deviation by applying a quadratic correction factor calculated from a Fis1ΔTM standard curve, (N = 2). Carbonate extraction buffer was 200 mM Na_2_CO_3_, pH 11.7.

### Circular Dichroism

Far UV Circular dichroism was performed on a Jasco J-720 circular dichroism spectropolarimeter with 5 µM Fis1ΔTM in reaction buffer with the indicated lipid vesicles, 25°C using a 1 mm pathlength, 3 accumulation average, with a scan rate of 20 nm/min, sensitivity of 20 mdeg, 2 second response from 190–260 nm. Samples were incubated for at least 2 hours before scanning at 25°C. A reference scan without protein in the presence and absence of lipids was subtracted to remove background ellipticity. Scans with vesicles alone were identical to buffer alone. Mean residue ellipticity ([Θ]residue, deg cm^2^ dmol^−1^) was calculated using the equation [Θ]_MRE_ = Θ/(10*[ Fis1ΔTM]*.1 cm)/(# of Residues). The fraction helicity was calculated according to the method outlined by Luo and Baldwin [41].

### Steady-State Fluorescence Spectroscopy and Acrylamide Quenching

Steady-state fluorescence measurements were done on a SLM-48000 spectrofluorometer (SLM-AMINCO, Urbana, IL). A 5 mm cuvette was used with 5 µM Fis1ΔTM (10 µM Trp) in reaction buffer. Excitation was at 295 nm (2 nm slit width) and emission at 310–370 nm (16 nm slit width). Polarizers were set at 90 degrees to reduce vesicle scatter. Spectra were baseline corrected using an identical sample that lacked protein. For quenching studies, acrylamide (Fisher Scientific) at the indicated concentration was added 5 minutes before the scan and the resulting data fit to the Stern-Volmer equation, F_0_/F = 1+*K*_sv_[Q], where F_0_ is the fluorescence in the absence of the quencher, F is the fluorescence in the presence of the quencher, *K*_sv_ is the Stern-Volmer quenching constant and [Q] is the concentration of quencher. Best fit lines were calculated using Igor Pro (Wavemetrics, Portland OR).

### Dynamic Light Scattering

Vesicle sizes were measured using dynamic light scattering on a Zetasizer nano ZS90 instrument equipped with a 633 nm laser, (Malvern). The dispersant was 425 µL of reaction buffer with 20 mM potassium acetate, 20 mM potassium phosphate using a calculated viscosity of .8910 cP and refractive index of 1.330. Before each measurement the sample was equilibrated at 25°C for 2 minutes. Each measurement was an average of 10 runs of 15 seconds and three measurements were averaged to give a final mean Z-average (Z-avg). All measurements were analyzed using the Dispersion Technology Software (Malvern) using the cumulants analysis on Multiple Narrow Mode. All samples collected had a polydispersity index (PDI) less than 0.2, indicating that the vesicles sizes were found to be monomodal. Protein alone under these conditions did not scatter enough light for the cumulants analysis.

### Electron Microscopy

Samples were prepared by mixing Fis1ΔTM and LUVs at the indicated concentration in reaction buffer at the designated pH. Copper formvar coated grids (Electron Microscopy Science) were ionized and then floated on 10 µL of samples for 5 minutes. The grids were rinsed quickly in deionized water, then imbedded into a 0.2% methylcelluose, 3.2% polyvinyl alcohol, 0.4% uranyl acetate solution. Images were taken on a Philips EM 420 TEM at 100 kV equipped with a SIS Megaview III CCD digital camera (Olympus).

### Light Scattering Kinetics by UV/Vis Spectrophotometry

Vesicles were added to a final concentration as noted to a 200 µL blanked buffer solution at either pH 5.0 or pH 7.0, mixed and the OD was measured on a Nanodrop 2000c UV/Vis spectrophotometer (Thermo Scientific) using a 1 cm pathlength cuvette, until the values reached equilibrium. At 450 nm, the absorbance due to the aromatic residue signal from the protein is negligible and the majority of the signal is due to light scattered at angles other than 180 degrees. For the experiment, the absorbance at 450 nm was measured every 30 seconds. An initial baseline was made with lipids and buffer alone at time 0. At approximately 1000 sec, 5 µM Fis1ΔTM (∼500 µM stock concentration, <1% volume change) was added, mixed by pipette and continued recording without missing a time point. At 4000 sec the solution was titrated either from pH 5.0 to pH 7.4 using a 4 M solution of fresh KOH (<1% volume change) or from pH 7.0 to pH 4.8 with HCl (<1% volume change) and recorded until 7000 sec time point. The pH of samples were confirmed using a pH meter to be within 0.5 a pH unit of the target pH.

### Kinetics of Vesicle Clustering

To estimate the rate equation for Fis1-induced vesicle clustering the kinetics at high vesicle∶protein ratios were measured under conditions in which vesicle collisions were not rate-limiting for clustering (up to 200 protein molecules per vesicle). At the higher protein concentrations, the vesicle concentration is effectively removed from the rate equation:
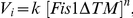
where *V_i_* is the initial velocity of the reaction, *k* is the apparent rate constant, [*Fis1ΔTM*] is the concentration of Fis1ΔTM and *n* is the order of the pseudo-rate constant. By measuring the initial velocity as a function of Fis1ΔTM concentration, the data can be fitted to provide an estimate of the value of *n*.

The experiments were performed with 100 µM 60∶40 DOPC∶DOPG (the highest lipid concentration to still give a measurable scattering signal at these ratios) in 20 mM potassium phosphate, 20 mM potassium acetate at pH 5.0 with stirring. The association reaction was started by addition of Fis1ΔTM in 20 µL of buffer to reach a final concentration that ranged from 1 nM to 200 nM in a 2 ml final volume. Scattering of each sample was measured by monitoring the absorbance at 333 nm at 10 second intervals with a 1 cm pathlength on a Nanodrop 2000c using cuvette mode (Thermo Scientific). This sampling rate was insufficient to reasonably fit the data at protein concentrations greater than 200 nM. For each measurement, the sample was blanked with lipid and buffer only and incubated for 5 minutes to establish a baseline reading. The absorbance (OD) versus time (seconds) was fit to a single exponential function to determine the initial velocity of association for each Fis1 condition using the software Igor Pro (Wavemetrics, Portland OR). The *V_i_* reported is from 3 independent experiments. The *V_i_* was plotted as a function of [Fis1] and fit to the above rate equation for *n* = 1, 2, or 3.

## Results

### The Fis1 cytosolic domain binds vesicles with lipid compositions that mimic the mitochondrial outer membrane

**Figure 1:**
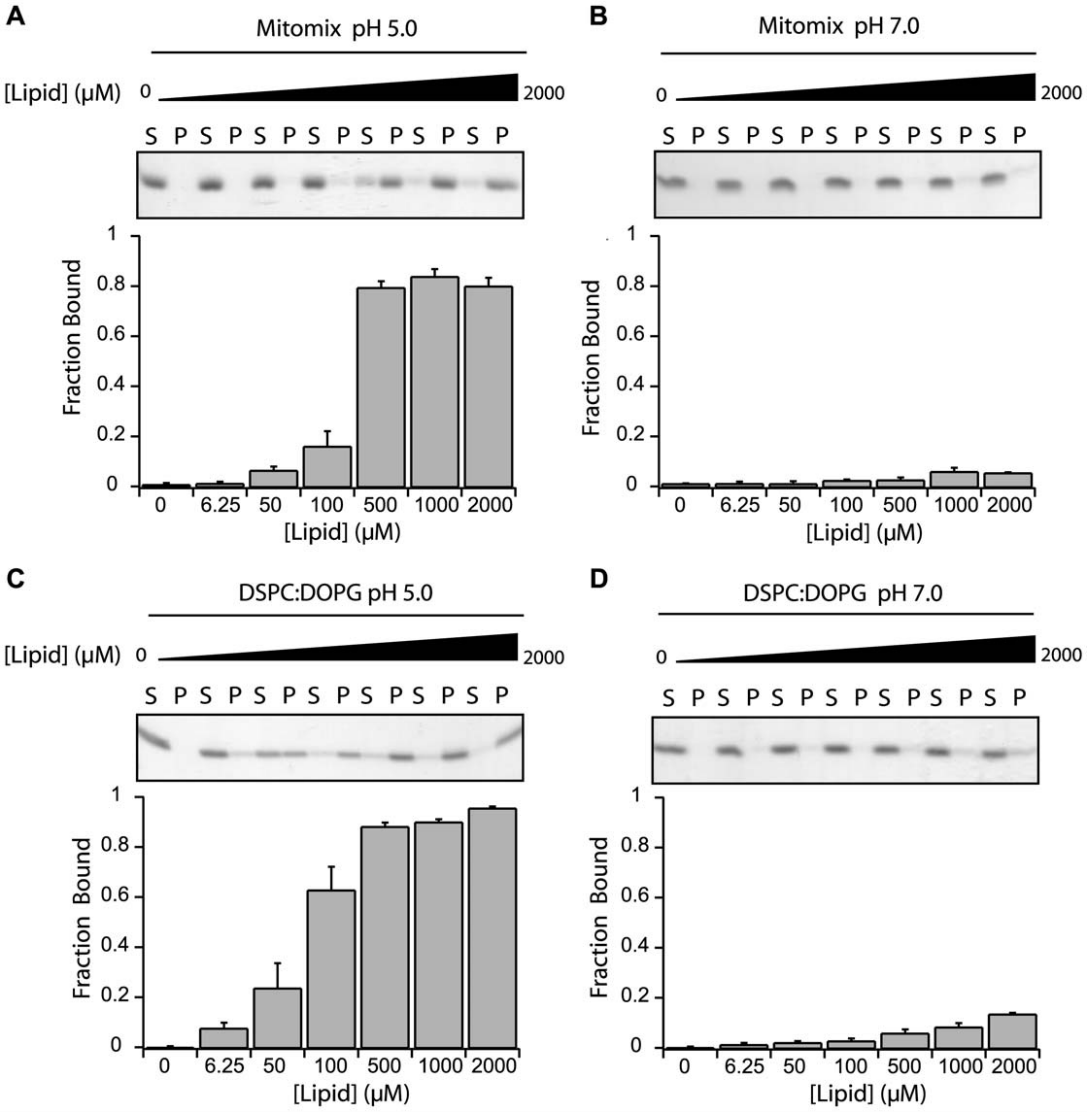
Fis1 lacking its transmembrane domain binds to membranes in a pH-dependent manner. A vesicle sedimentation assay was used to measure the fraction of Fis1ΔTM bound as a function of increasing lipid concentration for Mitomix lipids at pH 5.0(A) or pH 7.0(B) or Br_4_DSPC∶DOPG lipids at pH 5.0(C) or pH 7.0(D). Each panel shows SDS-PAGE analysis showing supernatant(S) and pellet(P) for increasing vesicle concentrations (above) and quantified by densitometry (below). Fis1ΔTM (5 µM) was incubated for 4 hours at 25°C with indicated concentrations of 100 nm large unilamellar vesicles. Average fraction bound and standard error of the mean were calculated from n = 3 independent measurements, except n = 5 in (D).

Previously the cytosolic domain of Fis1 (Fis1ΔTM) was shown to induce permeabilization of the small molecule ANTS from DOPC∶DOPG (6∶4) lipid vesicles at pH 5.0, but not at pH 7.0 [26]. To better understand this permeabilization, we first tested whether Fis1ΔTM could bind to lipid vesicles that mimic the mitochondrial outer membrane (Mitomix) [37]. In a vesicle sedimentation assay, we measured the amount of Fis1ΔTM bound to Mitomix lipid vesicles as a function of increasing lipid concentrations (Figure 1). At pH 5.0, Fis1ΔTM binding increased in a sigmoidal manner and saturated near 80% protein bound (1∶100 protein∶lipid) (Figure 1A). At pH 7.0, with the highest lipid concentration tested (1∶400, P∶L), ∼5% Fis1ΔTM bound to vesicles (Figure 1B). By contrast using a similar composition of lipid vesicles to that used in our earlier work (Br_4_DSPC∶DOPG,6∶4), we found a similar trend. At pH 5.0, Fis1ΔTM bound to a greater extent than the Mitomix vesicles and reached saturation at approximately 90% bound, (Figure 1C). At pH 7.0, with the highest lipid concentration tested (1∶400, P∶L), the interaction was ∼15% bound (Figure 1D). We consistently observed this increase in protein binding to Br_4_DSPC∶DOPG compared to Mitomix vesicles, which may arise from an increase in the amount of negatively charged lipid head groups compared to Mitomix vesicles (20% anionic headgroup for Mitomix vs. 40% for Br_4_DSPC∶DOPG mixture).

### Electrostatic forces stabilize membrane partitioning of Fis1ΔTM

**Figure 2:**
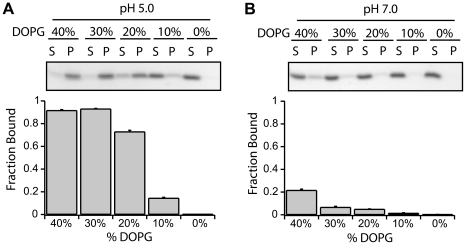
Fis1ΔTM membrane binding requires negatively charged lipids. A vesicle sedimentation assay was used to measure fraction of Fis1ΔTM bound to lipid vesicles as a function of decreasing concentration of anionic DOPG at pH 5.0(A) or pH 7.0(B). Each panel depicted as in Figure 1 with experiments done in a similar manner with 5 µM protein incubated for 4 hours at 25°C with 2 mM of 100 nm lipid vesicles (1∶400 protein∶lipid). Vesicles were made with a constant percentage 60% Br_4_DSPC, and DOPG was replaced by an equimolar amount of DOPC to maintain a constant lipid concentration. Average fraction bound and the standard error of the mean were calculated from n = 3 independent measurements.

**Figure 3:**
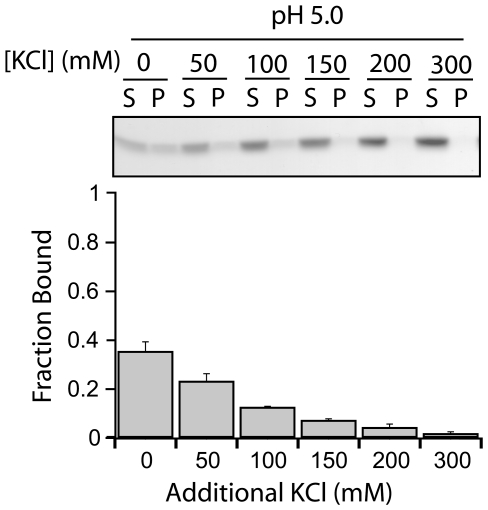
Fis1ΔTM membrane binding is salt dependent. A vesicle sedimentation assay was used to measure the fraction of Fis1ΔTM bound to lipid vesicles as a function of increasing ionic strength. Panel depicted as in Figure 1 with experiments done in a similar manner with 5 µM protein incubated for 6 hours at 25°C with 100 µM of 100 nm lipid vesicles (1∶20 protein∶lipid). Average fraction bound and the standard error of the mean were calculated from n = 2 independent measurements.

To test the effect of electrostatic forces on this interaction, we titrated the negatively charged lipid, DOPG, from 40% to 0% while maintaining the total lipid concentration using DOPC, and measured fraction protein bound. At both pH 5.0 and 7.0, decreasing the amount of negatively charged lipid (DOPG) decreased the amount of Fis1ΔTM bound (Figure 2). At pH 5.0, the largest decrease occurred between 20 to 10% DOPG (Figure 2A), whereas the largest decrease at pH 7.0 occurred from 40% to 30% DOPG (Figure 2B). These data indicate an electrostatic contribution to vesicle binding that arises from both the negative charge of the vesicles and from protonation of ionizable groups on Fis1ΔTM at pH 5.0. Altering the DOPE concentration did not affect Fis1ΔTM membrane binding (*data not shown*) suggesting that neutral lipids had little affect on binding. Consistent with this interpretation, the fraction of Fis1ΔTM bound to Mitomix vesicles (∼80% bound at P∶L of 1∶400 with 20% anionic content in Figure 1A) is similar to the fraction of Fis1ΔTM bound to PC/PG vesicles with the same ratio of negatively charged lipids (20% DOPG). The pH and anionic lipid dependence to Fis1 vesicle binding indicated a strong electrostatic contribution to the interaction. To further test this idea, we measured Fis1ΔTM binding to PC/PG membranes as a function of increasing concentration of KCl and observed a strong attenuation of binding at high salt (Figure 3). At 300 mM KCl, Fis1 binding was barely detectable in the vesicle fraction. This result was not a function of K^+^ because Na^+^ had a similar effect *(data not shown)*. Since neither KCl nor NaCl significantly affects Fis1ΔTM structure or stability, we interpret these data to indicate that membrane binding is favored by electrostatic interactions between the anionic headgroups of the lipid bilayer and the protein. Because the lipid headgroups are not thought to be titrated from pH 7.0 to 5.0 [42], [43], we attribute this affect to protonation of ionizable groups on Fis1.

### Fis1ΔTM is amphitropic and binds membranes reversibly

**Figure 4:**
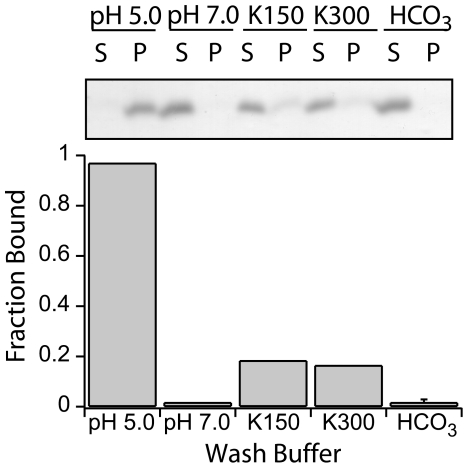
Fis1ΔTM membrane interaction is reversible. A vesicle sedimentation assay was used to measure reversibility of Fis1ΔTM bound to lipid vesicles. 5 µM Fis1ΔTM was incubated with 100 µM lipids (1∶20 protein∶lipid) at pH 5.0, centrifuged, resuspended in the indicated buffers: a pH 5.0 buffer control (pH 5.0), buffer at pH 7.0 (pH 7.0), buffer at pH 5.0 with an additional 150 mM KCl (K150), buffer pH 5.0 with an additional 300 mM KCl (K300), or 200 mM Na_2_CO_3_ pH 11.7 (HCO_3_). Resuspended vesicles were then analyzed for fraction bound as shown previously. Panel depicted as in Figure 1.

An important consideration of the Fis1-membrane interaction is whether Fis1ΔTM binds reversibly to membranes as a peripheral membrane protein, or is more characteristic of a permanently associated integral membrane protein. Therefore, we tested whether the Fis1ΔTM-membrane interaction is reversible by pH change, ionic strength change, and carbonate extraction. In these experiments, we bound protein to membrane vesicles (pH 5.0, 1∶20, P∶L), collected the lipid vesicles by sedimentation, resuspended in the indicated wash buffer, incubated for 2 hours, and determined the fraction bound. As expected, resuspension of pelleted vesicles with Fis1ΔTM in pH 5.0 buffer left all the protein in the lipid vesicle fraction (Figure 4, *pH 5.*0). In contrast, upon resuspension in pH 7.0 buffer, little protein was left in the lipid fraction (Figure 4, *pH 7.0*). This finding is similar to our initial binding results without first pre-binding Fis1ΔTM to the membranes (Figure 1) and shows that the pH-dependence of Fis1ΔTM binding to membrane vesicles is reversible. Increasing concentrations of KCl also resulted in partitioning of protein into the soluble fraction from the lipid fraction, (Figure 4, *K150 and K300*). A typical test to determine whether a protein interacts peripherally with the membrane is a carbonate extraction experiment, which resulted in <5% of protein in the vesicle fraction (Figure 4, *HCO_3_*). Together these results are consistent with the cytosolic domain of Fis1 binding to membranes in a manner that resembles a peripherally bound protein.

### Fis1ΔTM undergoes a conformational change upon membrane binding

**Figure 5:**
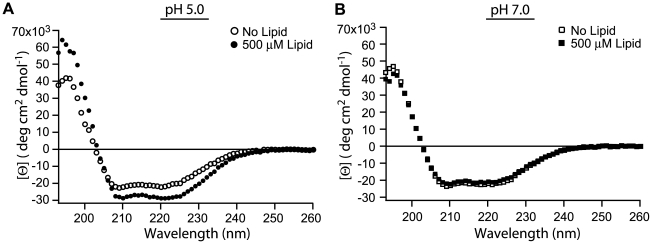
The α-helicity of Fis1ΔTM increases upon membrane binding. Circular dichroism spectropolarimetry was used to determine the mean residue ellipticity ([Θ]) of Fis1ΔTM in the presence (▪) or absence (□) of lipid vesicles at pH 5.0(A) and in the presence (•) or absence (○) of lipid vesicles at pH 7.0(B). For these experiments, 5 µM of protein was incubated for at least 2 hours at 25°C with 500 µM lipid vesicles (1∶100 protein∶lipid).

Reversible protein-membrane interactions are often associated with conformational changes of the protein from the soluble conformation to a membrane-bound conformation [29]. To assess this, we measured Far-UV CD spectropolarimetry of Fis1ΔTM in the presence and absence of lipid vesicles. In the absence of vesicles, the spectra are typical of well-folded α-helical proteins, with minima at 208 and 222 nm at both pH 7.0 and pH 5.0 (Figure 5). Although slow-timescale dynamics differ for regions of Fis1 between these two pH values [44], pH 7.0 and 5.0, CD spectra showed little differences (Figure 5A and 5B). Upon addition of 500 µM lipid vesicles at pH 5.0, the α-helical signal increased indicating an increase in secondary structure upon vesicle binding (Figure 5A). Using the method of Luo and Baldwin, vesicle binding increased the Fis1ΔTM helicity by 20%, from 58 to 78% [41]. No change was found after addition of vesicles at pH 7.0 (Figure 5B).

### Membrane binding buries Fis1ΔTM tryptophan residues

**Figure 6:**
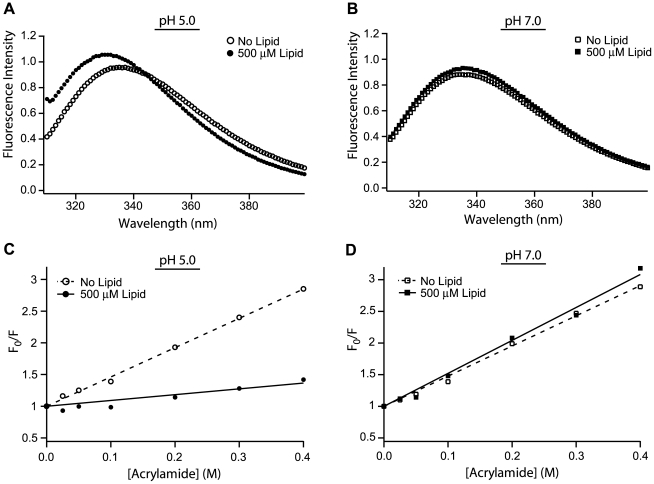
Tryptophan 7 and 47 of Fis1ΔTM are more protected from solvent upon membrane binding. Tryptophan fluorescence emission spectra were collected for Fis1ΔTM in the presence (▪) or absence (□) of lipid vesicles at pH 5.0(A) and in the presence (•) or absence (○) of lipid vesicles at pH 7.0(B). Quenching of intrinsic tryptophan fluorescence of Fis1ΔTM as a function of increasing concentration of the soluble quencher acrylamide in the presence (▪) or absence (□) of lipid vesicles at pH 5.0(C) and in the presence (•) or absence (○) of lipid vesicles at pH 7.0(D). The data were fit to the Stern-Volmer equation and best fit lines were plotted for no lipids (——) or 500 µM lipids (- - -). For these experiments 5 µM of Fis1ΔTM was incubated for at least 2 hours at 25°C with 500 µM lipid vesicles (1∶100 protein∶lipid).

Changes in tertiary structure upon membrane binding were assessed by intrinsic tryptophan fluorescence in the presence and absence of lipid vesicles. Fis1ΔTM contains two tryptophans that are in close proximity to each other and are partially protected from bulk solvent in the NMR structural ensemble (1y8m.pdb) [20]. Tryptophan 7 lies in the N-terminal arm that is thought to block access to a concave binding surface that includes tryptophan 47 and other evolutionarily conserved residues [25]. At pH 5.0 in the presence of 500 µM lipids (1∶100, P∶L), the fluorescence signal increased significantly and was accompanied by a blue shift in λ_max_ by 6 nm (Figure 6A). At pH 7.0 in the presence of 500 µM lipids, the fluorescence signal slightly increased with little change in λ_max_ consistent with the conditions where we found <10% of Fis1ΔTM bound (Figure 6B). While changes in fluorescence intensity are difficult to interpret, the blue shift in λ_max_ upon membrane binding indicates a change in the Trp environment that is more non-polar.

In order to compare the solvent accessibility between the solution and membrane-bound conformations of Fis1ΔTM, we measured the Stern-Volmer quenching coefficient (*K*_SV_) for acrylamide, a water-soluble quencher of intrinsic tryptophan fluorescence. The acrylamide quenching data were linear at both pH 5.0 and pH 7.0 (Figure 6C and 6D) indicating that the two tryptophans in Fis1ΔTM have similar solvent accessibilities. This linearity also allows the data to be fit to determine *K*_SV_. At pH 5.0 and 7.0 in the absence of vesicles, we found that Fis1ΔTM tryptophans are equally protected with *K*_SV_ values of 4.62 M^−1^ at pH 5.0 and 4.77 M^−1^ at pH 7.0. The *K*_SV_ value provides a measure of solvent accessibility and these values are typical for solvent exposed to moderately exposed tryptophans in soluble proteins [45]–[47]. By contrast, under conditions in which Fis1ΔTM robustly binds vesicles at pH 5.0, the *K*_SV_ value decreased significantly to 0.91 M^−1^ (from 4.62 M^−1^) indicating that Fis1ΔTM tryptophans are significantly more protected from solvent upon membrane binding (Figure 6C). In the presence of lipid vesicles at pH 7.0, we found only a slight increase in protection consistent with the intrinsic tryptophan fluorescence data (Figure 6D). We interpret the fluorescence and circular dichroism data collectively to indicate that Fis1ΔTM undergoes a dramatic structural rearrangement upon membrane binding that results in burial of its tryptophan residues while retaining its secondary structure. We also note that this conformational change is completely reversible (*data not shown*).

### The cytosolic domain of Fis1 affects membrane structure

**Figure 7:**
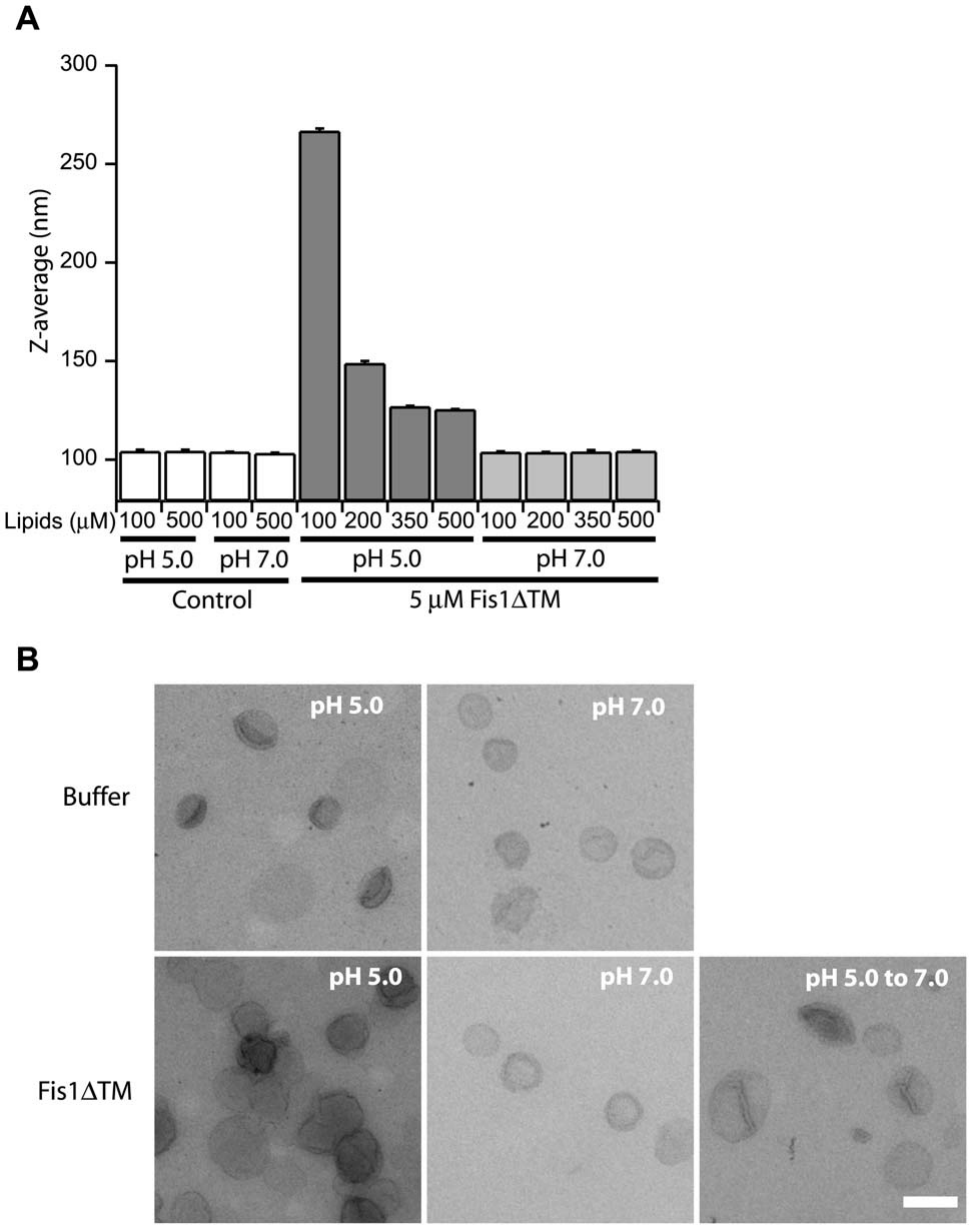
Fis1ΔTM affects vesicle size and shape at pH 5.0, but not pH 7.0. Lipid vesicle size was determined in the presence and absence of Fis1ΔTM at pH 5.0 or pH 7.0 by dynamic light scattering(A) and negative stain electron microscopy(B). Vesicle size is reported by Z-average values for dynamic light scattering in (A) and the scale bar is 200 nm in (B). For these experiments, 100 nm lipid vesicles were incubated for 2 hours at 25°C with buffer at pH 7.0, pH 5.0 or with 5 µM Fis1ΔTM at pH 7.0, or pH 5.0 (1∶20 protein∶lipid). To assess vesicle size upon returning to pH 7.0, vesicles were incubated in the presence of 5 µM Fis1ΔTM at pH 5.0 for 1 hour, then a 50× pH 7.0 buffer was added to adjust the pH to 7.0, (15% change in volume). Samples were incubated for 1 additional hour at pH 7.0 and then put on grids and imaged.

Fis1 is proposed to mediate membrane fission by recruiting the dynamin-like mechanoenzyme, Dnm1, to sites of fission. During endocytosis, dynamin mediates membrane scission along with other molecules that also alter membrane structure and are thought to be important for this process [48], [49]. Given the reversible, membrane-induced conformational change of Fis1, we asked whether Fis1 itself might alter membrane structure. To test this idea, we used dynamic light scattering to determine the size distribution of lipid vesicles in the presence and absence of Fis1ΔTM and as a function of increasing lipid concentration. Incubation in the absence of protein had no effect on vesicle size distribution over a 24 hr period, with a mean Z-average of 104±1 nm, PDI = 0.17 (Figure 7A, *control*). At pH 5.0, Fis1ΔTM incubation increased the Z-average size of the vesicles in all conditions tested, with the highest protein∶lipid ratios giving the species with the greatest apparent size (Figure 7A, *pH 5.0*). The PDI for these samples remained less than 0.2 indicating a homogenous population of vesicle sizes. At pH 7.0, Fis1ΔTM incubation resulted in no significant change in the Z-average of the vesicles at any of the protein to lipid ratios tested (mean Z-avg 104±1 nm) (Figure 7A, *pH 7.0*), even after 24 hours. These data indicate a pH-dependent increase in light scattering mediated by the Fis1 cytosolic domain upon addition to lipid vesicles.

We next used negative staining electron microscopy to assess the origin of increased light scattering. Vesicles in the absence of protein at pH 5.0 or pH 7.0 were indistinguishable except for a slightly darker staining at pH 5.0. At a 1∶40 protein∶lipid ratio, we found large aggregates that appeared composed of clustered vesicles that retained their original spherical shape (Figure 7B). Unlike protein-mediated vesicle fusion [50], we found no evidence for large spherical vesicles. Our results are inconsistent with Fis1ΔTM mediating a simple fusion of membranes, and are more consistent with closely apposed membranes that maintain the original curvature of the 100 nm vesicle. At lower protein∶lipid ratios large clusters still formed, but at a much lower frequency (<1% of total vesicles). Vesicles incubated with protein at pH 7.0 were indistinguishable from vesicle-alone controls (Figure 7B), consistent with the light scattering data, which indicates that significant binding of Fis1 is necessary for vesicle clustering.

The conclusions from electron microscopy were confirmed using fluorescence light microscopy of 100 nm vesicles that have been doped with the fluorescent lipid Rh-DOPE. Under similar conditions as before (pH 5.0, 1∶20, P∶L), larger particles (>1 µm) were visible after 5–10 seconds and grew with time (*data not shown*). Vesicles appeared to cluster in a random manner, often forming inhomogeneous strings of vesicles or large vesicular clumps. Incubation overnight or longer, resulted in clusters visible to the naked eye. At all times, the most abundant and largest complexes were seen at the highest protein∶lipid ratios. Vesicle clustering also occured at lower protein∶lipid ratios but was less frequent and resulted in smaller aggregates.

**Figure 8:**
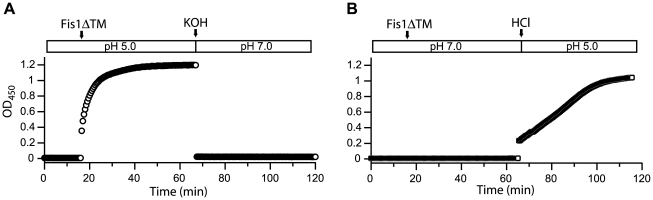
Fis1ΔTM induces rapid reversible vesicle clustering. The ability of Fis1ΔTM to alter lipid vesicle size as assessed by light scattering as a function of time upon deprotonation(A) or protonation(B). 100 nm lipid vesicles were added to a final concentration of 100 µM to a blanked buffer solution at either pH 5.0(A) or pH 7.0(B) until the signal reached equilibrium. Absorbance at 450 nm was then measured every 30 seconds as a measure of light scattering. At 17 minutes, 5 µM Fis1ΔTM was added to the solution (1∶20 protein∶lipid), mixed and the next 30 second time point was taken. At 67 min, either 4 M KOH was added to titrate the solution from pH 5.0 to pH 7.0(A) or 4 M HCl was added to titrate the solution from pH 7.0 to pH 5.0(B). Each reagent addition changed the volume less than 1%.

Given that Fis1ΔTM binding to lipid vesicles is reversible, we asked if the Fis1-induced membrane clustering was also reversible. We first induced clustering at pH 5.0 by adding Fis1ΔTM at a 1∶40 protein∶lipid ratio. This sample after 30 minutes was opaque to the naked eye indicating vesicle clustering. After 1 hour, we titrated this solution from pH 5.0 to pH 7.0, incubated for 1 hour and then analyzed the vesicle population by negative-stain EM. Surprisingly, we found that the vesicles returned to their original size and shape (Figure 7b). To determine the time-dependence of vesicle clustering, we monitored light scattering as measured by a change in optical density at 450 nm (OD_450_), where the OD_450_ of the solution is proportional to vesicle clustering. At pH 5.0, protein addition to vesicles (P∶L, 1∶20) dramatically increased the OD_450_ signal (Figure 8A). Upon addition of concentrated base to bring the pH to 7.0, the OD_450_ signal precipitously fell to values similar to those in the absence of protein (Figure 8A, *KOH arrow*). Since little Fis1ΔTM is bound under these conditions at pH 7.0, we interpret these data to indicate a reversal of Fis1-induced vesicle clustering within 30 sec. Analysis of these vesicles by dynamic light scattering gave a Z-average of 164 nm (*data not shown*). However, this value is skewed by a small number of larger vesicles that remain clustered (PDI = 0.254 and >2000 nm as determined by DLS and EM). These data indicated that clustered vesicles predominantly returned to their original size distribution. We next pre-incubated Fis1ΔTM with vesicles at pH 7.0 and found no change to the OD_450_ signal, consistent with our earlier observations of little Fis1ΔTM bound under these conditions (Figure 8B). By contrast, acidification of this solution to pH 5.0 increased the OD_450_ in a near linear fashion (Figure 8B, *HCl arrow*). Similar titrations of protein or vesicle samples alone did not change the OD_450_ signal (*data not shown*). The rate of vesicle clustering in these two experiments differed, which likely arises from differences in local protein concentration that may indicate a requirement for protein oligomerization.

**Figure 9:**
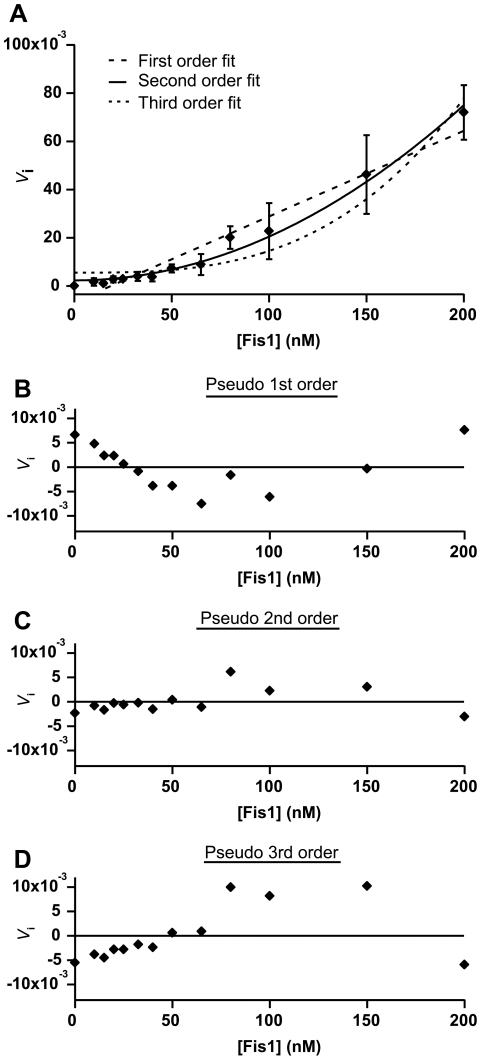
At least two Fis1ΔTM molecules are required for vesicle clustering. The initial velocity of vesicle clustering was measured under conditions that are independent of vesicle concentration to determine its dependence on Fis1ΔTM concentration. (A) The initial velocity (*V*_i_) obtained was plotted as a function of [Fis1ΔTM] and fit to a pseudo-first (— —), second (—) or third (- - -) order rate law. Residuals to the fit for pseudo-first (B), second (C) and third (D) order are shown. The data is best fit to a pseudo-second order rate law with an overall apparent rate constant of *k* = 1.82×10^−6^ nM^−1^ s^−1^ and a coefficient of determination of R^2^ = 0.9866. For comparison, the pseudo-first order fit gave *k* = 3.55×10^−4^ s^−1^ with R^2^ = 0.9525) and the pseudo-third order fit gave *k* = 9.05×10^−9^ nM^−2^ s^−1^ and R^2^ = 0.9285.

To assess whether Fis1 oligomerization is involved in vesicle clustering, we conducted further kinetic measurements at a constant lipid concentration (100 µM) that was not rate-limiting for clustering ([Lipid]≫[Protein]). We assumed under these conditions, that the initial rate of clustering is primarily dependent on the concentration of protein (*see materials and methods*). We measured the initial velocity of clustering at a constant vesicle concentration as a function of increasing concentrations of Fis1ΔTM to determine the overall order of the reaction, which is related to the number of Fis1 molecules required for each clustering event. We found that the initial velocity increased in a non-linear manner when plotted against [Fis1ΔTM] (Figure 9A) and was best fit to a reaction rate model that was pseudo-second order in Fis1 concentration with an overall apparent rate constant of *k* = 1.82×10^−6^ nM^−1^ s^−1^ and a coefficient of determination of R^2^ = 0.9866. By comparison, reaction rate models that were either pseudo-first order or pseudo-third order gave poor fits to the data (R^2^ = 0.9525 and R^2^ = 0.9285, respectively, Figure 9B–D). These data are consistent with at least two Fis1 molecules being necessary for vesicle clustering.

## Discussion

In this study, we show that the cytosolic domain of fission protein, Fis1, undergoes a membrane-induced conformational change that results in lipid vesicle clustering. This clustering does not result from membrane fusion, but is reversible and vesicles largely return to identical size and shape. This reversibility is rapid and the Fis1-membrane association is favored by a high protein∶lipid ratio. Whether vesicle clustering is required for organelle fission is not known. However, many proteins involved in membrane dynamics mediate a close membrane association for their cellular function [51]. For membrane fission, the mechanics of each scission event requires the close apposition of two bilayers. In order to complete scission, each of the apposing bilayers must fuse adjacent to the site of scission. Thus each membrane scission event requires two membrane fusion events and would likely be aided by proteins that could promote reversible membrane association [52]. Our data leads us to speculate that vesicle clustering induced by the Fis1 cytosolic domain might play a role in organelle fission.

Several issues need to be addressed for this speculation to be correct. First, we expect vesicle clustering to be specific to Fis1. Although visible aggregation between positively charged proteins and negatively charged membranes has been reported [53], [54], the structure-based PROPKA method [55] estimates the mean charge of Fis1 at pH 5.0 to be −1. Therefore, it is unlikely that Fis1 associates with negatively charged membranes using a simple mechanism of net positive charge, especially given the random distribution of charges in its structure [20]. The conformational change upon membrane binding is also greater than one might expect from a non-specific interaction. Notably, the soluble domains of other tail-anchored proteins, Bcl-xL and CED-9, have not induced vesicle clustering in similar experiments to those presented here [38], [56], [57], supporting the idea that reversible vesicle clustering may be specific to Fis1.

The apparent requirement for protonation to induce vesicle clustering may seem unlikely *in vivo*, but two considerations may address this concern. First, Fis1 does indeed bind to membranes at pH 7.0, though weakly, and this interaction could be enhanced by other factors that are not present in our experiments. For instance, the cytosolic domain is normally tethered to the mitochondrial outer membrane by its C-terminal transmembrane domain and this tethering likely enhances the protein-membrane interaction significantly [58]. This tethering may also reduce the apparent need for protonation in clustering vesicles, as has been shown for the tail-anchored Bcl-2 protein, Bcl-xL [38]. However, for Fis1 we have been unable to test this idea because the full-length molecule has proven difficult to isolate and to reconstitute into membranes in a form similar to the solution structure. Second, it may be that the effective pH at the surface of intracellular membranes is lower than the cytosol. This idea is based on recent evidence that a negatively charged membrane surface, such as that found on peroxisomes and mitochondria, has an effective pH that is lower than bulk solution [59]–[63]. This effective decrease in local pH at the surface of these membranes would be expected to promote the Fis1-membrane interaction. In addition, other factors, such as change in the molecular environment around the membrane or membrane curvature may also be important for inducing a Fis1-membrane interaction.

The high concentrations of Fis1 necessary for vesicle clustering might also seem unlikely, *in vivo*. Fis1 is thought to be distributed uniformly on the surface of mitochondria [2], but recruits proteins specifically to sites of scission. Therefore a physiological, yet unknown, mechanism exists to determine a site of scission, which may involve assembling Fis1 molecules. Our data supports at least two Fis1 molecules being necessary for vesicle clustering. Previously, mammalian Fis1 has been shown to oligomerize in cross-linking and blue-native PAGE experiments [64], [65] and the cytosolic domain of human Fis1 crystallized as a dimer [66]. Whether Fis1 from budding yeast oligomerizes *in vivo* is not known, however, we have found that it dimerizes in solution upon recombinant expression *(unpublished data)*. This raises the possibility that Fis1 could act in a SNARE-like manner to tether two vesicles together, although our data cannot exclude the possibility of dimerization on the same vesicle or two molecules that remain independent. This work lays the foundation for identifying mutations in Fis1 that disrupt vesicle clustering, possibly by disrupting dimerization. Such mutagenesis studies will be useful for determining the mechanism of clustering and its physiological role.

Reversible protein-membrane interactions are well-appreciated in the regulation of many cellular processes [27]–[29]. These processes often involve amphitropic domains that exist in both distinct soluble and membrane bound conformations, similar to the cytosolic domain of Fis1. Amphitropic proteins often utilize a conformational change as a result of a cellular signal to regulate their biological activities [67], [68]. In one case similar to that reported here, the amphitropic nature of creatine kinase may be oligomerization-dependent in a manner important for mitochondrial inner membrane morphology [69]. More generally, our work also highlights a different kind of protein amphitropism that involves transmembrane-anchored proteins, which play important roles in a variety of biological processes [37], [70]–[72]. The human genome is estimated to contain approximately 400 tail-anchored proteins [37], [72]. Given the ability of the transmembrane domain to increase the effective concentration of the cytosolic domain at the membrane, it is reasonable to ask whether the cytosolic domains of these proteins undergo reversible membrane interactions. Indeed, such interactions are evolutionarily conserved between the pro- and anti-apoptotic Bcl-2 proteins where a critical event appears to be the permeabilization of the mitochondrial outer membrane by the cytosolic domain of pro-apoptotic Bax [73]–[78]. Tail-anchored proteins, such as these, must have evolved mechanisms distinct from other proteins that either prevent (non-amphitropic) or promote (amphitropic) interactions with the membrane. Such mechanisms are currently unknown, and difficult to measure *in vivo*, but our work here suggests that controlling the electrostatic forces of these interactions may be critical.

In conclusion, we have shown that a single protein domain has the ability to mediate dramatic changes in membrane structure through reversible clustering. We speculate that the Fis1-membrane interaction is important in mediating membrane association that occurs during membrane fission.

## References

[1] Osteryoung KW, Nunnari J (2003). The division of endosymbiotic organelles.. Science.

[2] Mozdy AD, McCaffery JM, Shaw JM (2000). Dnm1p GTPase-Mediated Mitochondrial Fission is a Multi-Step Process Requiring the Novel Integral Membrane Component Fis1p.. J Cell Biol.

[3] Tieu Q, Nunnari J (2000). Mdv1p is a WD Repeat Protein that Interacts with the Dynamin-Related GTPase, Dnm1p, to Trigger Mitochondrial Division.. J Cell Biol.

[4] Cerveny KL, McCaffery JM, Jensen RE (2001). Division of Mitochondria Requires a Novel DMN1-Interacting Protein, Net2p.. Mol Biol Cell.

[5] Fekkes P, Shepard KA, Yaffe MP (2000). Gag3p, an outer membrane protein required for fission of mitochondrial tubules.. J Cell Biol.

[6] Yoon Y, Krueger EW, Oswald BJ, McNiven MA (2003). The mitochondrial protein hFis1 regulates mitochondrial fission in mammalian cells through an interaction with the dynamin-like protein DLP1.. Mol Cell Biol.

[7] Otsuga D, Keegan BR, Brisch E, Thatcher JW, Hermann GJ (1998). The Dynamin-Related GTPase, Dnm1p, Controls Mitochondrial Morphology in Yeast.. J Cell Biol.

[8] Smirnova E, Shurland DL, Ryazantsev SN, van der Bliek AM (1998). A human dynamin-related protein controls the distribution of mitochondria.. J Cell Biol.

[9] Motley A, Ward GP, Hettema E (2008). Dnm1p-dependent peroxisome fission requires Caf4p, Mdv1p and Fis1p.. J Cell Sci.

[10] Lackner LL, Horner JS, Nunnari J (2009). Mechanistic analysis of a Dynamin Effector.. Science.

[11] Chan DC (2006). Mitochondria: Dynamic Organelles in Disease, Aging, and Development.. Cell.

[12] Olichon A, Guillou E, Delettre C, Landes T, Arnauné-Pelloquin L (2006). Mitochondrial dynamics and disease, OPA1.. Biochim Biophys Acta.

[13] Leinninger GM, Edwards JL, Lipshaw MJ, Feldman EL (2006). Mechanisms of disease: mitochondria as new therapeutic targets in diabetic neuropathy.. Nat Clin Pract Neurol.

[14] Waterham HR, Koster J, van Roermund CW, Mooyer PA, Wanders RJ (2007). A lethal defect of mitochondrial and peroxisomal fission.. N Engl J Med.

[15] Stojanovski D, Koutsopoulos OS, Okamoto K, Ryan MT (2004). Levels of Human Fis1 at the Mitochondrial Outer Membrane Regulate Mitochondrial Morphology.. J Cell Sci.

[16] Suzuki M, Jeong SY, Karbowski M, Youle RJ, Tjandra N (2003). The Solution Structure of Human Mitochondria Fission Protein Fis1 Reveals a Novel TPR-Like Helix Bundle.. J Mol Biol.

[17] Griffin EE, Graumann J, Chan DC (2005). The WD40 Protein Caf4p is a Component of the Mitochondrial Fission Machinery and Recruits Dnm1p to Mitochondria.. J Cell Biol.

[18] Schauss AC, Bewersdorf J, Jakobs S (2006). Fis1p and Caf4p, but Not Mdv1p, Determine the Polar Localization of Dnm1p Clusters on the Mitochondrial Surface.. J Cell Sci.

[19] Karren MA, Coonrod EM, Anderson TK, Shaw JM (2005). The Role of Fis1p-Mdv1p Interactions in Mitochondrial Fission Complex Assembly.. J Cell Biol.

[20] Suzuki M, Neutzner A, Tjandra N, Youle RJ (2005). Novel Structure of the N Terminus in Yeast Fis1 Correlates with a Specialized Function in Mitochondrial Fission.. J Biol Chem.

[21] Zhang Y, Chan DC (2007). Structural Basis for Recruitment of Mitochondrial Fission Complexes by Fis1.. Proc Natl Acad Sci U S A.

[22] Koch A, Thiemann M, Grabenbauer M, Yoon Y, McNiven MA (2003). Dynamin-like protein 1 is involved in peroxisomal fission.. J Biol Chem.

[23] Bhar D, Karren MA, Babst M, Shaw JM (2006). Dimeric Dnm1-G385D interacts with Mdv1 on mitochondria and can be stimulated to assemble into fission complexes containing Mdv1 and Fis1.. J Biol Chem.

[24] Nagotu S, Krikken AM, Otzen M, Kiel JA, Veenhuis M (2008). Peroxisome fission in Hansenula polymorpha requires Mdv1 and Fis1, two proteins also involved in mitochondrial fission.. Traffic.

[25] Wells RC, Picton LK, Williams SC, Tan FJ, Hill RB (2007). Direct Binding of the Dynamin-Like GTPase, Dnm1, to Mitochondrial Dynamics Protein Fis1 is Negatively Regulated by the Fis1 N-Terminal Arm.. J Biol Chem.

[26] Fannjiang Y, Cheng WC, Lee SJ, Qi B, Pevsner J (2004). Mitochondrial Fission Proteins Regulate Programmed Cell Death in Yeast.. Genes Dev.

[27] Halskau O, Muga A, Martinez A (2009). Linking new paradigms in protein chemistry to reversible membrane-protein interactions.. Curr Protein Pept Sci.

[28] Hurley JH (2006). Membrane binding domains.. Biochim Biophys Acta.

[29] Johnson JE, Cornell RB (1999). Amphitropic proteins: regulation by reversible membrane interactions (review).. Mol Membr Biol.

[30] Cheever ML, Sato TK, de Beer T, Kutateladze TG, Emr SD (2001). Phox domain interaction with PtdIns(3)P targets the Vam7 t-SNARE to vacuole membranes.. Nat Cell Biol.

[31] Lee SA, Kovacs J, Stahelin RV, Cheever ML, Overduin M (2006). Molecular mechanism of membrane docking by the Vam7p PX domain.. J Biol Chem.

[32] Schiavo G, Gu QM, Prestwich GD, Sollner TH, Rothman JE (1996). Calcium-dependent switching of the specificity of phosphoinositide binding to synaptotagmin.. Proc Natl Acad Sci U S A.

[33] Wu Y, He Y, Bai J, Ji SR, Tucker WC (2003). Visualization of synaptotagmin I oligomers assembled onto lipid monolayers.. Proc Natl Acad Sci U S A.

[34] Frazier AA, Wisner MA, Malmberg NJ, Victor KG, Fanucci GE (2002). Membrane orientation and position of the C2 domain from cPLA2 by site-directed spin labeling.. Biochemistry.

[35] Arac D, Chen X, Khant HA, Ubach J, Ludtke SJ (2006). Close membrane-membrane proximity induced by Ca(2+)-dependent multivalent binding of synaptotagmin-1 to phospholipids.. Nat Struct Mol Biol.

[36] Kuwana T, Mackey MR, Perkins G, Ellisman MH, Latterich M (2002). Bid, Bax, and lipids cooperate to form supramolecular openings in the outer mitochondrial membrane.. Cell.

[37] Henderson MP, Billen LP, Kim PK, Andrews DW (2007). Cell-free analysis of tail-anchor protein targeting to membranes.. Methods.

[38] Thuduppathy GR, Craig JW, Kholodenko V, Schon A, Hill RB (2006). Evidence that membrane insertion of the cytosolic domain of Bcl-xL is governed by an electrostatic mechanism.. J Mol Biol.

[39] Wimley WC, Hristova K, Ladokhin AS, Silvestro L, Axelsen PH (1998). Folding of beta-sheet membrane proteins: a hydrophobic hexapeptide model.. J Mol Biol.

[40] Candiano G, Bruschi M, Musante L, Santucci L, Ghiggeri GM (2004). Blue silver: a very sensitive colloidal Coomassie G-250 staining for proteome analysis.. Electrophoresis.

[41] Luo P, Baldwin RL (1997). Mechanism of helix induction by trifluoroethanol: a framework for extrapolating the helix-forming properties of peptides from trifluoroethanol/water mixtures back to water.. Biochemistry.

[42] Tocanne JF, Teissie J (1990). Ionization of phospholipids and phospholipid-supported interfacial lateral diffusion of protons in membrane model systems.. Biochim Biophys Acta.

[43] Cevc G, Marsh D (1987). Phospholipid Bilayers:Physical Properties and Models.

[44] Picton LK, Casares S, Monahan AC, Majumdar A, Hill RB (2009). Evidence for conformational heterogeneity of fission protein Fis1 from Saccharomyces cerevisiae.. Biochemistry.

[45] Lakowicz JR (2006). Principles of Fluorescence Spectroscopy.

[46] Calhoun DB, Vanderkooi JM, Holtom GR, Englander SW (1986). Protein fluorescence quenching by small molecules: protein penetration versus solvent exposure.. Proteins.

[47] Merrill AR, Palmer LR, Szabo AG (1993). Acrylamide quenching of the intrinsic fluorescence of tryptophan residues genetically engineered into the soluble colicin E1 channel peptide. Structural characterization of the insertion-competent state.. Biochemistry.

[48] Hinshaw JE (2000). Dynamin and its role in membrane fission.. Annu Rev Cell Dev Biol.

[49] De Camilli P, Takei K, McPherson PS (1995). The function of dynamin in endocytosis.. Curr Opin Neurobiol.

[50] Stegmann T, Doms RW, Helenius A (1989). Protein-mediated membrane fusion.. Annu Rev Biophys Biophys Chem.

[51] Martens S, McMahon HT (2008). Mechanisms of membrane fusion: disparate players and common principles.. Nat Rev Mol Cell Biol.

[52] Chernomordik L, Kozlov MM, Zimmerberg J (1995). Lipids in biological membrane fusion.. J Membr Biol.

[53] Bergers JJ, Vingerhoeds MH, van Bloois L, Herron JN, Janssen LH (1993). The role of protein charge in protein-lipid interactions. pH-dependent changes of the electrophoretic mobility of liposomes through adsorption of water-soluble, globular proteins.. Biochemistry.

[54] Kim J, Kim H (1989). Penetration and fusion of phospholipid vesicles by lysozyme.. Arch Biochem Biophys.

[55] Li H, Robertson AD, Jensen JH (2005). Very fast empirical prediction and rationalization of protein pKa values.. Proteins.

[56] Thuduppathy GR, Terrones O, Craig JW, Basanez G, Hill RB (2006). The N-terminal domain of Bcl-xL reversibly binds membranes in a pH-dependent manner.. Biochemistry.

[57] Tan FJ, Zuckerman JE, Wells RC, Hill RB (2011). The C. elegans B-cell lymphoma 2 (Bcl-2) homolog cell death abnormal 9 (CED-9) associates with and remodels lipid membranes.. Protein Sci.

[58] Adam G, Delbruck M, Rich A, Davidson N (1968). Reduction of dimensionality in biological diffusion processes.. Structural Chemistry and Molecular Biology.

[59] Yamashita T, Voth GA (2010). Properties of hydrated excess protons near phospholipid bilayers.. J Phys Chem B.

[60] Branden M, Sanden T, Brzezinski P, Widengren J (2006). Localized proton microcircuits at the biological membrane-water interface.. Proc Natl Acad Sci U S A.

[61] Murray D, Ben-Tal N, Honig B, McLaughlin S (1997). Electrostatic interaction of myristoylated proteins with membranes: simple physics, complicated biology.. Structure.

[62] Ben-Tal N, Honig B, Peitzsch RM, Denisov G, McLaughlin S (1996). Binding of small basic peptides to membranes containing acidic lipids: theoretical models and experimental results.. Biophys J.

[63] Vaz WL, Nisksch A, Jahnig F (1978). Electrostatic interactions at charged lipid membranes. Measurement of surface pH with fluorescent lipoid pH indicators.. Eur J Biochem.

[64] Jofuku A, Ishihara N, Mihara K (2005). Analysis of functional domains of rat mitochondrial Fis1, the mitochondrial fission-stimulating protein.. Biochem Biophys Res Commun.

[65] Serasinghe MN, Yoon Y (2008). The mitochondrial outer membrane protein hFis1 regulates mitochondrial morphology and fission through self-interaction.. Exp Cell Res.

[66] Dohm JA, Lee SJ, Hardwick JM, Hill RB, Gittis AG (2004). Cytosolic Domain of the Human Mitochondrial Fission Protein Fis1 Adopts a TPR Fold.. Proteins.

[67] Yethon JA, Epand RF, Leber B, Epand RM, Andrews DW (2003). Interaction with a membrane surface triggers a reversible conformational change in Bax normally associated with induction of apoptosis.. J Biol Chem.

[68] Cornell RB (1991). Regulation of CTP:phosphocholine cytidylyltransferase by lipids. 1. Negative surface charge dependence for activation.. Biochemistry.

[69] Stachowiak O, Dolder M, Wallimann T (1996). Membrane-binding and lipid vesicle cross-linking kinetics of the mitochondrial creatine kinase octamer.. Biochemistry.

[70] Rabu C, Schmid V, Schwappach B, High S (2009). Biogenesis of tail-anchored proteins: the beginning for the end?. J Cell Sci.

[71] Borgese N, Brambillasca S, Colombo S (2007). How tails guide tail-anchored proteins to their destinations.. Curr Opin Cell Biol.

[72] Kutay U, Hartmann E, Rapoport TA (1993). A class of membrane proteins with a C-terminal anchor.. Trends Cell Biol.

[73] Hsu YT, Wolter KG, Youle RJ (1997). Cytosol-to-membrane redistribution of Bax and Bcl-X(L) during apoptosis.. Proc Natl Acad Sci U S A.

[74] Wolter KG, Hsu YT, Smith CL, Nechushtan A, Xi XG (1997). Movement of Bax from the cytosol to mitochondria during apoptosis.. J Cell Biol.

[75] Shimizu S, Narita M, Tsujimoto Y (1999). Bcl-2 family proteins regulate the release of apoptogenic cytochrome c by the mitochondrial channel VDAC.. Nature.

[76] Antonsson B, Montessuit S, Lauper S, Eskes R, Martinou JC (2000). Bax oligomerization is required for channel-forming activity in liposomes and to trigger cytochrome c release from mitochondria.. Biochem J.

[77] Minn AJ, Velez P, Schendel SL, Liang H, Muchmore SW (1997). Bcl-x(L) forms an ion channel in synthetic lipid membranes.. Nature.

[78] Basanez G, Zhang J, Chau BN, Maksaev GI, Frolov VA (2001). Pro-apoptotic cleavage products of Bcl-xL form cytochrome c-conducting pores in pure lipid membranes.. J Biol Chem.

